# Targeting Angiotensinogen With *N*-Acetylgalactosamine–Conjugated Small Interfering RNA to Reduce Blood Pressure

**DOI:** 10.1161/ATVBAHA.123.319897

**Published:** 2023-10-19

**Authors:** Dien Ye, Edwyn O. Cruz-López, Ho-Chou Tu, Ivan Zlatev, A.H. Jan Danser

**Affiliations:** 1 Division of Pharmacology and Vascular Medicine, Department of Internal Medicine, Erasmus MC, University Medical Center Rotterdam, The Netherlands (D.Y., E.O.C.-L., A.H.J.D.).; 2 Alnylam Pharmaceuticals, Cambridge, MA (H.-C.T., I.Z.).

**Keywords:** angiotensinogen, angiotensins, hepatocytes, hypertension, renin

## Abstract

Blood pressure management involves antihypertensive therapies blocking the renin-angiotensin system (RAS). Yet, it might be inadequate due to poor patient adherence or the so-called RAS escape phenomenon, elicited by the compensatory renin elevation upon RAS blockade. Recently, evidence points toward targeting hepatic AGT (angiotensinogen) as a novel approach to block the RAS pathway that could circumvent the RAS escape phenomenon. Removing AGT, from which all angiotensins originate, should prevent further angiotensin generation, even when renin rises. Furthermore, by making use of a trivalent *N*-acetylgalactosamine ligand–conjugated small interfering RNA that specifically targets the degradation of hepatocyte-produced mRNAs in a highly potent and specific manner, it may be possible in the future to manage hypertension with therapy that is administered 1 to 2× per year, thereby supporting medication adherence. This review summarizes all current findings on AGT small interfering RNA in preclinical models, making a comparison versus classical RAS blockade with either ACE (angiotensin-converting enzyme) inhibitors or AT1 (angiotensin II type 1) receptor antagonists and AGT suppression with antisense oligonucleotides. It ends with discussing the first-in-human study with AGT small interfering RNA.

**Highlights**

- Targeting hepatic angiotensinogen as a novel approach to block the renin-angiotensin system.
- Zilebesiran is an investigational *N*-acetylgalactosamine–conjugated small interfering RNA therapeutic that specifically targets human hepatic angiotensinogen mRNA. A phase 1 first-in-human study has revealed that zilebesiran is an effective strategy to lower blood pressure in patients with hypertension.
- This review addresses the mechanism of action of zilebesiran, making use of detailed animal studies comparing this novel drug with classical blockers of the renin-angiotensin system like ACE (angiotensin-converting enzyme) inhibitors and angiotensin receptor blockers. It also describes its safety profile.

Hypertension is one of the major preventable risk factors for cardiovascular diseases including ischemic heart disease, stroke, myocardial infarction, as well as other complications such as chronic kidney disease.^1^ Since the renin-angiotensin system (RAS) is central in blood pressure regulation, several approved antihypertensive therapies target this pathway. Despite the availability of effective antihypertensive agents, a large proportion of patients with hypertension still do not achieve guideline-recommended blood pressure levels, and thus hypertension remains an area of high unmet need.^2^

Please see www.ahajournals.org/atvb/atvb-focus for all articles published in this series.

Possible reasons for inadequate blood pressure management under the current RAS-blocking antihypertensive therapies include poor patient adherence and the so-called RAS escape phenomenon elicited by the compensatory renin elevation upon RAS blockade.^3,4^ This would result in upregulated angiotensin formation, allowing the (partial) return of Ang II (angiotensin II)–induced effects, including its effects on aldosterone release.^5^ Poor adherence or nonadherence might result in huge costs, given that additional investigations will be needed to assess why there is no response.

Recently, preclinical and clinical evidence points toward targeting hepatic AGT (angiotensinogen) as a novel approach to block the RAS pathway that could circumvent the RAS escape phenomenon.^6–11^ AGT is the sole substrate for renin cleavage, that is, the first enzymatic step of the RAS cascade, which eventually leads to the production of Ang II in the circulation and peripheral tissues to activate its AT_1_ (Ang II type 1) receptor. AT_1_ receptor activation results in a number of downstream physiological events, most notably vasoconstriction and a rise in blood pressure. By depleting AGT production, one could envision that Ang II will also eventually be depleted even in the presence of compensatory upregulation of renin, thus circumventing the RAS escape phenomenon. AGT mRNA and protein are primarily produced by the hepatocytes in the liver, although expression of AGT is also claimed in extrahepatic tissues such as the kidney, brain, heart, and adipose tissue.^12–15^ Data on extrahepatic production are often conflicting, for instance, first claiming that adipocyte-specific AGT deletion reduced plasma AGT by 25%,^16^ and then suggesting that adipose-specific AGT deficiency does not affect plasma AGT levels.^17^ Yet, preclinical studies support that plasma and tissue Ang II are solely derived from liver AGT protein.^7–10,15,18^ Hepatic AGT is secreted into the circulation and subsequently enters peripheral tissues, either by diffusion^19^ or via receptor-mediated cellular uptake.^20^ Therefore, hepatocyte targeting of AGT depletion becomes an attractive strategy to block the RAS pathway, as it can circumvent the RAS escape phenomenon.

## LIVER TARGETING OF AGT WITH *N*-ACETYLGALACTOSAMINE SMALL INTERFERING RNA AND OTHER mRNA-TARGETING THERAPIES

Trivalent *N*-acetylgalactosamine (GalNAc) ligand–conjugated small interfering RNA (siRNA) represents an emerging therapeutic modality that specifically targets the degradation of hepatocyte-produced mRNAs in a highly potent and specific manner. The ASGR (asialoglycoprotein receptor), also known as the Ashwell-Morell receptor, is expressed on hepatocytes and facilitates uptake and clearance of circulating glycoproteins with exposed terminal galactose and GalNAc glycans via clathrin-mediated endocytosis.^21^ The architectural design of this trivalent GalNAc ligand,^21^ when attached to a chemically modified siRNA,^22^ allows for highly efficient hepatocyte uptake through interaction with the ASGR, followed by cell internalization of both the receptor and ligand with the siRNA cargo (Figure). This ASGR-mediated delivery system for GalNAc-siRNA conjugates currently represents one of the state-of-the-art modalities in the therapeutic development of investigational oligonucleotides as clinical candidates.^23^ Likewise, there are currently 4 Food and Drug Administration–approved GalNAc-siRNA therapeutics: GIVLAARI (givosiran),^24^ OXLUMO (lumasiran),^25^ LEQVIO (inclisiran),^26^ and AMVUTTRA (vutrisiran),^27^ targeting the hepatocyte-specific expression of 5-aminolevulinic acid synthase 1, glycolate oxidase 1, PCSK9 (proprotein convertase subtilisin kexin type 9), and transthyretin, respectively. Givosiran is used in patients with acute hepatic porphyria, lumasiran in patients with primary hyperoxaluria type 1, inclisiran in patients with hypercholesterolemia, and vutrisiran in patients with polyneuropathy of hereditary transthyretin-mediated amyloidosis.

**Figure.**
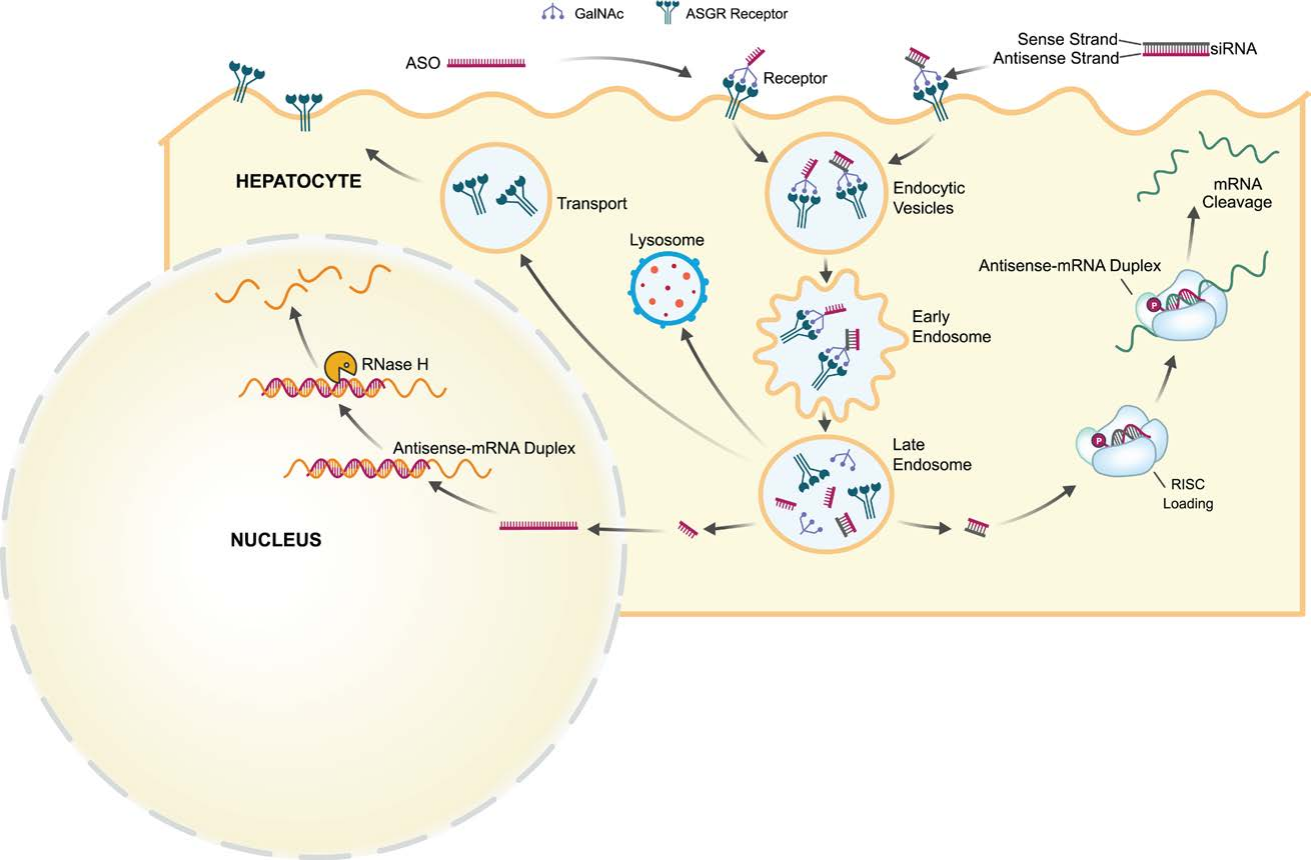
**Overview of the proposed mechanism of hepatocyte uptake of *N*-acetylgalactosamine (GalNAc) small interfering RNA (siRNA) and GalNAc antisense oligonucleotide (ASO) conjugates by ASGR (asialoglycoprotein receptor)-mediated endocytosis.** Intracellular uptake by harnessing the ASGR results in the accumulation of the siRNAs and ASOs in the late endosomes, where they can reside for a prolonged period of time. Slow release of the siRNA from the late endosomes into the cytosol triggers loading of the siRNA in the RNA-induced silencing complex (RISC), which ensures hybridization to the target mRNA and subsequent mRNA cleavage and degradation. Slow release of the ASO from the late endosomes into the cytosol allows for further trafficking of the ASO inside the cell nucleus, which ensures hybridization to the target mRNA and recruitment of the RNase H nuclease for subsequent mRNA cleavage and degradation.

While the GalNAc ligand is the chemistry component that allowed for oligonucleotide delivery to hepatocytes, the evolution of RNA interference (RNAi) therapeutics has been driven by advances in siRNA chemical modification designs.^23^ Whereas the first generation of GalNAc-siRNA conjugates required relatively high-dose levels and higher dosing frequencies to afford the desired in vivo pharmacological activity,^28^ advancements of the chemical modification patterns led to the enhanced stabilization chemistry siRNA designs, which significantly improved potency and duration compared with the first generation,^21,22^ resulting in effective RNAi-mediated mRNA silencing in hepatocytes with much lower dose levels across species, including humans. Potency improvement in vivo is attributed by the refinement of siRNA chemistry, in particular by optimizing the positions of 2′-deoxy-2′-fluoro and 2′-*O*-methyl ribosugar modifications, as well as phosphorothioate backbone modifications across both sense and antisense strands of the double-stranded siRNA duplex to enhance stability without compromising its intrinsic RNAi activity.^22^ Advanced designs with low 2′-deoxy-2′-fluoro content that yield further improved potency and duration in preclinical species, including nonhuman primates,^29^ have been linked to higher liver drug levels, indicating that the improvement in potency is predominantly due to increased metabolic stability of the siRNA conjugates in vivo.

Another hallmark of the GalNAc-siRNA conjugates is the long durability of silencing that can persist for several months after a single-dose administration in preclinical species and also humans.^28–31^ Mechanistically, the increased metabolic stability of the chemically modified siRNA molecules in the hepatocyte acidic intracellular compartments (eg, the endolysosomal system) contributes to the prolonged duration and activities in vivo.^32^ It has been demonstrated that functional siRNAs can be liberated from these compartments over time and loaded into the Argonaute 2 protein in the cytoplasm, a key step to form a functional RNA-induced silencing complex, which ensures hybridization of the siRNA guide strand with the target mRNA and mediates mRNA cleavage and degradation (Figure).^32^ A previous report^32^ highlighted that due to the chemical modifications mentioned above, the chemically modified siRNAs are able to maintain prolonged stability in these acidic intracellular compartments, thus enabling the continuous generation of functional RNA-induced silencing complexes and continuous RNAi activity over a long period of time (weeks to months), providing evidence that the intracellular acidic compartments serve as a long-term depot for GalNAc-siRNA conjugates.^32^ In summary, the metabolically stable, chemically modified, enhanced stabilization chemistry GalNAc-siRNAs,^21,22^ combined with the durable intracellular depot, are the major contributor to the extended duration of activity observed in vivo. The long-duration pharmacokinetic and pharmacodynamic properties may provide the potential to apply infrequent dosing schedules (biannually or even annually in some cases) and could potentially address therapy nonadherence.

More recently, a further chemical nucleotide modification has been reported that enhances the clinical safety of GalNAc-siRNA conjugates by reducing the potential for off-target mRNA binding. This strategy utilizes siRNA guide strand seed-pairing destabilization and has demonstrated its benefit both in preclinical species and in clinical development.^33^ In this context, a single glycol nucleic acid or a 2′-5′-RNA modification reduces siRNA seed-mediated Watson and Crick base pair hybridization binding to off-target transcripts while maintaining the on-target RNAi activity. In siRNAs with established hepatotoxicity driven by off-target effects, these novel designs with seed-pairing destabilization, termed enhanced stabilization chemistry plus, demonstrated an improved therapeutic window in rats.^33,34^ It is also noted that recent clinical data from multiple investigational GalNAc-siRNA therapeutics supported acceptable long-term safety profiles of the GalNAc-siRNA platform, suggesting a potential to utilize this technology more broadly to treat prevalent diseases in the general population.^31^

Another class of therapeutic oligonucleotides, antisense oligonucleotides (ASOs), has also benefited from the introduction of the GalNAc ligand for effective delivery to hepatocytes.^35^ Second-generation ASOs promote degradation of complementary mRNA via the RNase H mechanism (Figure).^36^ Similar to GalNAc-siRNAs, a majority of the target mRNAs for ASOs are predominantly expressed in hepatocytes, which makes the ASGR-GalNAc targeting strategy particularly advantageous.^37^ There are currently no clinically approved GalNAc-ASOs. However, there are over 10 different ongoing clinical trials.^38,39^

## ANIMAL STUDIES WITH AGT GalNAc-siRNA AND OTHER mRNA-TARGETING THERAPIES: COMPARISON VERSUS CLASSICAL RAS BLOCKERS

We and others have investigated the GalNAc-siRNA strategy as an antihypertensive approach in multiple preclinical hypertension rodent models (Table), including the spontaneously hypertensive rat (SHR), the 5/6th nephrectomy Sprague-Dawley (SD) rat,^10^ the deoxycorticosterone acetate (DOCA)-salt–treated SD rat, and the diabetic TGR(mRen2)27 rat.^15^ The latter rat overexpresses mouse renin and thus represents a model of Ang II–dependent hypertension. It was found that effective blood pressure lowering is achieved when >95% of AGT is knocked down.^7–10,15^

**Table.**
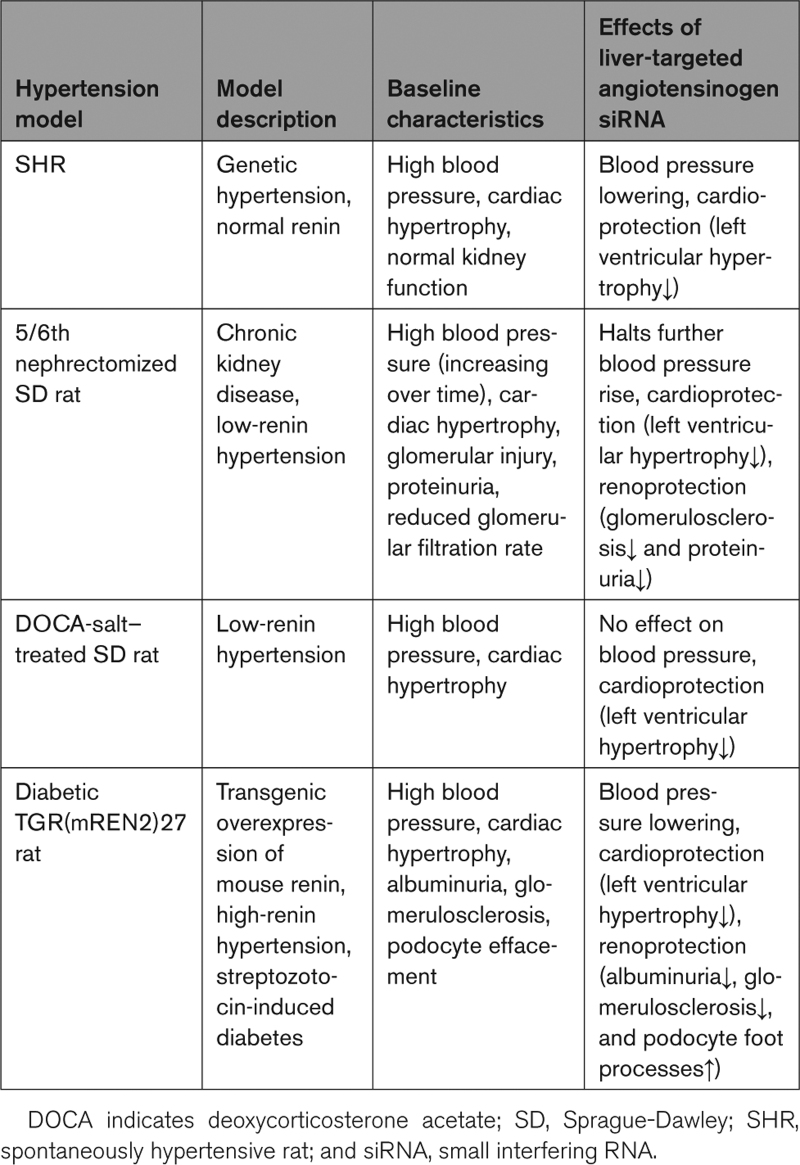
Overview of the Findings Made With Angiotensinogen siRNA in Various Hypertensive Rat Models^7,8,10,15^

Not surprising, in the DOCA-salt model, where circulating renin is greatly suppressed, no blood pressure–lowering effect of AGT siRNA was observed, while mineralocorticoid receptor blockade with spironolactone did normalize blood pressure.^8^ The latter is to be expected, since DOCA acts as a mineralocorticoid receptor agonist. Yet, in this model, upregulated brain RAS activity has also been proposed to underlie the hypertension.^40^ From this perspective, central RAS blockade would be required for a blood pressure–lowering effect. Although liver-targeted AGT siRNA did not affect brain AGT synthesis, it did lower brain Ang II levels, indicating that brain angiotensin generation in this model, if occurring, depends on hepatic AGT. The disappearance of brain Ang II combined with a lack of a blood pressure–lowering effect after AGT siRNA raises doubt about the concept of selective brain RAS upregulation in the DOCA-salt model (involving local AGT synthesis in the brain) as the cause of hypertension, also because the amount of renin in brain tissue is low and at most represents circulating renin in trapped blood.^41^ A more simple explanation is that brain Ang II is taken up from blood.^42^ Interestingly, despite the absence of a blood pressure–lowering effect, AGT siRNA did reduce cardiac hypertrophy in the DOCA-salt rat. Since this was paralleled by a reduction in cardiac Ang II, it most likely represents the fact that AGT siRNA is capable of blocking Ang II formation at cardiac tissue sites. Clearly, such formation also depends on hepatic AGT.^43,44^

In the SHR, the degree of blood pressure lowering by AGT siRNA was similar to that of classical RAS blockers including ACE (angiotensin-converting enzyme) inhibitors and ARBs (AT_1_ receptor blockers), while combining GalNAc-AGT siRNA and an ARB resulted in synergistic and superior blood pressure lowering (≈70 versus ≈20–25 mm Hg for each drug separately).^7^ This might be the consequence of further renin upregulation during combined RAS blockade, resulting in the virtual disappearance of AGT. The latter resembles the situation in patients with heart failure who are being treated with multiple RAS blockers.^45,46^ Their renin levels are so high that they induce AGT depletion. Indeed, when combining GalNAc-AGT siRNA and an ARB, circulating AGT levels in the SHR were reduced by 99.8% (versus 97.9% with AGT siRNA alone) and circulating Ang II virtually disappeared. Greatly diminishing Ang II production plus simultaneously blocking the AT_1_ receptor (no longer allowing even the small quantities of Ang II that might still be present to stimulate their receptor) appeared to be a powerful combination that brought RAS activity to almost zero.^47^ These data strongly support the concept that circulating AGT is liver derived, in full agreement with data in liver AGT knockout animals.^17,48^ They also raise the question to what degree AGT production at extrahepatic sites plays a role. Surprisingly, although GalNAc-AGT siRNA did not affect the AGT mRNA levels at extrahepatic sites (including the kidney and adipose tissue),^7–9^ it did greatly diminish the AGT protein levels at these sites. Apparently, therefore, despite local AGT expression, the actual AGT protein at these extrahepatic sites is liver derived. Given these findings, which coincide with observations in transgenic animals genetically lacking kidney or adipose tissue AGT,^20,49–52^ currently, mechanisms are being investigated that might explain AGT uptake from blood. Here, an important newly identified candidate is the megalin receptor.^20,53–56^ Indeed, without this receptor, renal angiotensin content was greatly reduced, both under normal and disease conditions.^20,57^ Another candidate, at least in the heart, is low-density lipoprotein receptor–related protein 1.^58^ Taken together, it appears that tissue production of angiotensins depends on liver-derived AGT. This concerns virtually every known site of local angiotensin production, that is, heart, vascular wall, adipose tissue, kidney, and brain. Future studies should reevaluate whether local AGT expression outside the liver truly has any role. Importantly, in the SHR AGT, siRNA did lower cardiac Ang II and cardiac hypertrophy.^7^ However, the main determinant of the latter appeared to be the reduction in blood pressure, rather than the disappearance of cardiac Ang II.

In the 5/6th nephrectomy SD rat, blood pressure slowly rises over time. GalNAc-AGT siRNA, when given after 5 weeks, prevented a further rise, yet did not normalize blood pressure. This effect was similar to that of combined ACE inhibitor plus ARB treatment.^10^ The modest blood pressure–lowering effect of RAS blockade in this hypertensive model most likely reflects the fact that it is a low-renin model. In fact, the animals were unable to upregulate renin during RAS blockade. This contrasts greatly with the SHR. Nevertheless, AGT siRNA offered renoprotection in this model (a reduction in glomerulosclerosis and diminished proteinuria), and this involved the suppression of renal Ang II independently of any blood pressure–lowering effect.

Similarly, GalNAc-AGT siRNA offered renoprotection in the diabetic TGR(mRen2)27 rat. Streptozotocin-induced diabetes in this rat model resulted in albuminuria, accompanied by glomerulosclerosis and podocyte effacement, without a change in the glomerular filtration rate. RAS blockade with either the ACE inhibitor captopril, the ARB valsartan, or GalNAc-AGT siRNA lowered blood pressure identically, and modestly larger drops in blood pressure were observed when combining GalNAc-AGT siRNA and valsartan or captopril and valsartan. This differs from the synergistic antihypertensive effects of dual RAS blockade observed in the SHR and suggests that the high blood pressure in this model is largely the consequence of mouse renin overexpression and not related to diabetes per se. In other words, as soon as blood pressure is back to normal (by blocking the effects of mouse renin overexpression), more RAS blockade does not yield substantial further blood pressure lowering. All treatments lowered cardiac hypertrophy. Given the strong correlation between cardiac hypertrophy and blood pressure, the main underlying mechanism for this effect appeared to be the decrease in blood pressure. Although AGT siRNA did not affect renal AGT mRNA expression, confirming its liver specificity, it did lower renal AGT levels. Furthermore, only AGT siRNA with or without valsartan lowered renal angiotensin I. All treatments lowered renal Ang II, and the reduction was the largest (>95%) in the AGT siRNA+valsartan group (>95%, versus ≈40% after AGT siRNA alone). All treatments identically lowered albuminuria. Yet, only AGT siRNA with or without valsartan restored podocyte foot processes and reduced glomerulosclerosis. These data, therefore, suggest that the renoprotective effects of AGT siRNA reflect interference with renal Ang II generation, in a blood pressure–independent manner.

The ASO approach targeting AGT was capable of lowering circulating AGT in SHR to a similar degree as AGT siRNA.^6,7^ Interestingly, non–GalNAc-conjugated ASOs additionally suppressed extrahepatic AGT expression, for example, in kidney and adipose tissue, while GalNAc conjugation, as expected, exclusively resulted in hepatic AGT suppression.^6^ Yet, non–GalNAc-conjugated and GalNAc-conjugated AGT identically lowered blood pressure, and the degree of blood pressure lowering correlated with the degree of circulating AGT reduction. The maximum hypotensive effect persisted until day 10, in agreement with the fact that the ASO liver half-life is 2 to 4 weeks. AGT ASO suppression has also been evaluated in 8% salt-fed SHR (a low-renin model),^6^ and here, its antihypertensive effect was comparable to the combined effect of captopril and losartan, while captopril alone was ineffective. Only AGT ASO lowered aldosterone in the 8% salt-fed SHR, and this aldosterone reduction may have contributed to its blood pressure–lowering effect. When exposing salt-depleted SD rats (a high-renin model) to either non–GalNAc-conjugated ASOs or GalNAc-conjugated ASOs, it was observed that only the non–GalNAc-conjugated ASOs resulted in histological alterations in the kidney, like tubular degeneration and thickening of the glomerular basement membrane.^6^ Such alterations were also observed with the ARB losartan, and when treating the 5/6th nephrectomy SD rat with a non–GalNAc-conjugated ASO, a GalNAc-conjugated ASO, or the ACE inhibitor captopril, it was again observed that only the non–GalNAc-conjugated ASO and the classical RAS blocker captopril induced histological alterations.^6^ These alterations have been suggested to rely on the multiclonal expansion and transformation of renin cells from a classical endocrine phenotype to a matrix-secretory phenotype.^59^ Based on these observations, it was argued that non–GalNAc-conjugated ASOs and classical RAS blockers result in excessive RAS suppression in the kidney (which massively upregulates renin), thereby eventually inducing renal damage, while GalNAc-conjugated ASOs would not do this since they leave renal AGT intact. Here, it is important to realize that full suppression of the RAS, particularly at the level of the kidney, is not desirable, given that normal renal function is RAS dependent.^5^ Hence, too much RAS blockade might result in renal damage due to the absence of renal Ang II. Unfortunately, the authors did not report renal Ang II levels after ASO exposure, and thus, to what degree GalNAc-conjugated ASOs truly left renal Ang II in the normal range (by not interfering with renal AGT expression) is unknown. They did observe reduced renal AGT levels after exposure to GalNAc-conjugated ASOs, suggesting that also in their hands, at least part of renal AGT was liver derived. Moreover, rises in plasma renin were higher with the non–GalNAc-conjugated ASOs than with the GalNAc-conjugated ASOs. This latter finding supports that the RAS blockade with the non–GalNAc-conjugated ASOs was more potent, and thus an alternative explanation of the data is that the hepatic effects of the non–GalNAc-conjugated ASOs were stronger than those of the GalNAc-conjugated ASOs. This explanation does not require the concept of ongoing renal Ang II generation from locally synthesized AGT. Indeed, near-complete suppression of hepatic AGT synthesis with liver-targeted AGT siRNA has already been reported to abolish renal Ang II.^10,15,60^

In summary, in a variety of animal models, GalNAc-AGT siRNA alone or in combination with an ARB resulted in renal and cardiac protective benefits accompanied by consistent lowering of local tissue Ang II levels in both blood pressure–dependent and blood pressure–independent manners.^7–10,15^ It is thought that lowering of tissue (especially renal and cardiac) Ang II levels by GalNAc-AGT siRNA is a key contributor to the overall therapeutic benefits seen in animal models in addition to its effect in systemic blood pressure lowering. Observations with GalNAc-AGT ASO were largely similar to those with the siRNA approach.

## HUMAN STUDIES WITH AGT siRNA

Zilebesiran is an investigational GalNAc-siRNA therapeutic that specifically targets human hepatic AGT mRNA and eventually inhibits the production of AGT protein. A multipart phase 1 first-in-human study (https://www.clinicaltrials.gov; NCT03934307) of zilebesiran was conducted in patients with hypertension.^30^ Patients received single ascending subcutaneous doses of zilebesiran (10–800 mg) or placebo. Compared with placebo, zilebesiran was associated with dose-dependent reductions (up to >95% in high-dose levels) in serum AGT. Moreover, single doses of zilebesiran (≥200 mg) resulted in reductions in systolic (>10 mm Hg) and diastolic (>5 mm Hg) blood pressure by week 8 post-treatment, and the zilebesiran-mediated blood pressure reductions were consistent throughout the 24-hour diurnal cycle and sustained to 24 weeks. The effects of zilebesiran 800 mg on blood pressure were also assessed under low/high-sodium diet conditions and with irbesartan coadministration. Blood pressure reductions upon zilebesiran treatment were attenuated by high-salt diet and augmented with irbesartan coadministration. The phase 1 results provide strong support that hepatic AGT targeting using an RNAi approach may be an effective strategy to lower blood pressure in humans. Importantly, single doses of zilebesiran up to 800 mg were well tolerated in patients, with no observation of hypotension, hyperkalemia, or worsening renal function requiring medical intervention up to 24 weeks after dosing. Consistent with the levels of hepatic protein lowering observed in other therapies that utilize the GalNAc-siRNA platform,^26,27,61^ zilebesiran demonstrated prolonged pharmacodynamic profile of serum AGT lowering and sustained reduction of blood pressure up to 24 weeks. Two follow-on phase 2 studies for zilebesiran are also underway: KARDIA-1 (https://www.clinicaltrials.gov; NCT04936035) is a randomized double-blind dose-ranging study evaluating the efficacy and safety of zilebesiran in patients with mild-to-moderate hypertension, and in KARDIA-2 (https://www.clinicaltrials.gov; NCT05103332), the efficacy and safety of zilebesiran on blood pressure will be investigated in combination with 3 different classes of conventional antihypertensives with distinct mechanisms of action.

## SAFETY OF AGT siRNA

A potential benefit of AGT siRNA over currently available antihypertensive drugs may be better treatment adherence because of the low frequency at which siRNA needs to be dosed. Additionally, this approach offers continuous and durable RAS blockade, as opposed to varying RAS blockade when making use of drugs that are dosed daily. Yet, acute RAS activation may be needed to maintain adequate arterial pressure and tissue perfusion during certain hypotensive medical scenarios such as shock or emergency surgery. In rodent models, AGT siRNA-mediated blood pressure lowering can be rapidly reversed by administration of Ang II or norepinephrine, or gradually reversed by fludrocortisone or high-salt intake,^9^ suggesting that conventional vasopressors may be an intervention that can address emergency hypotensive episodes. However, clinical data are needed to evaluate whether results seen in preclinical studies hold in humans.

AGT levels rise during pregnancy (up to 2-fold to 3-fold).^62^ Pregnancy demands major cardiovascular, renal, and endocrine changes to provide an adequate blood supply for the growing fetus. Since the RAS plays a key role in this adaptation process, as well as in fetal growth (eg, in renal development),^48,63^ RAS blockers are contraindicated in pregnancy. This likely also applies to AGT siRNA. Nevertheless, future studies should address to what degree it passes the placenta and whether it might be used for the treatment of preeclampsia.^64^

Given the liver accumulation of the siRNA, one might speculate about liver toxicity or inflammatory and immunologic side effects. Yet, no such observations were made for liver-targeted PCSK9 siRNA (inclisiran) over a 6-month period.^31^ Indeed, with a number of approved clinical products, and with a substantial body of preclinical investigative work,^29,34,65^ it is now well established that liver-targeting GalNAc-siRNA conjugates have favorable preclinical safety profiles and wide therapeutic windows. Furthermore, liver enzyme data in diabetic TGR(mRen2)27 rats did not show any liver deterioration after AGT siRNA treatment, either alone or in combination with valsartan.^15^

## CONCLUSIONS

The GalNAc-siRNA therapeutic is a novel approach to lower hepatic AGT. In the abovementioned animal studies, hepatic AGT-lowering approaches were associated with blood pressure–lowering effects at least comparable to those of classical RAS blockers like ACE inhibitors and ARBs, and when combined with such blockers, synergy may occur. The first data in humans thus far align with this view. Additionally, preclinical evidence suggests that this approach may result in both renoprotection and cardioprotection. AGT siRNA predominantly acts at the tissue level, illustrating the fact that tissue angiotensin production relies on hepatic AGT. Theoretically, it allows the full disappearance of circulating AGT, but the desired degree of RAS suppression is unlikely to be 100%, since the RAS is needed for normal kidney function and the maintenance of blood pressure, for example, during shock. Using a GalNAc-siRNA approach for the treatment of hypertension may yield a future therapeutic regimen that is administered 1 to 2× per year, thereby potentially improving medication adherence. Nevertheless, this also poses a threat in situations where the RAS is acutely needed, and thus preclinical and clinical programs are now carefully investigating its efficacy and safety profile. Finally, to what degree the beneficial effects of AGT suppression also involve long-term suppression of aldosterone still needs to be investigated. Short-term studies in animals support that its effects on aldosterone are comparable to those of classical RAS blockers.^7,15^

## ARTICLE INFORMATION

### Acknowledgments

The authors thank Petya Zlateva for the graphical artwork on the Figure.

### Sources of Funding

None.

### Disclosures

E.O. Cruz-López was supported by the Mexican National Council of Science and Technology (grant No. 739513). I. Zlatev and H.-C. Tu are employees and stockholders of Alnylam Pharmaceuticals. A.H.J. Danser received a grant from Alnylam Pharmaceuticals, which has partially supported the work described in this review. The other author reports no conflicts.
